# Prominent role of histone lysine demethylases in cancer epigenetics and therapy

**DOI:** 10.18632/oncotarget.24319

**Published:** 2018-01-25

**Authors:** Avilala Janardhan, Chandrasekhar Kathera, Amrutha Darsi, Wajid Ali, Lingfeng He, Yanhua Yang, Libo Luo, Zhigang Guo

**Affiliations:** ^1^ The No. 7 People's Hospital of Changzhou, Changzhou, China; ^2^ Jiangsu Key Laboratory for Molecular and Medical Biotechnology, College of Life Sciences, Nanjing Normal University, Nanjing, China

**Keywords:** epigenetics, histone modifications, protein methylation, lysine specific demethylases, therapeutics

## Abstract

Protein methylation has an important role in the regulation of chromatin, gene expression and regulation. The protein methyl transferases are genetically altered in various human cancers. The enzymes that remove histone methylation have led to increased awareness of protein interactions as potential drug targets. Specifically, Lysine Specific Demethylases (LSD) removes methylated histone H3 lysine 4 (H3K4) and H3 lysine 9 (H3K9) through formaldehyde-generating oxidation. It has been reported that LSD1 and its downstream targets are involved in tumor-cell growth and metastasis. Functional studies of LSD1 indicate that it regulates activation and inhibition of gene transcription in the nucleus. Here we made a discussion about the summary of histone lysine demethylase and their functions in various human cancers.

## INTRODUCTION

Epigenetics refers to heritable changes regulating gene expression that are not a result of changes in the primary DNA sequence. In cancer, aberrant epigenetic silencing of tumour suppressor genes is a common occurrence that is associated with abnormal DNA methylation patterns and changes in covalent histone modifications. Histone proteins are subjected to post-translational modifications, like acetylation, methylation, phosphorylation, and ubiquitination, and these histone modifications act as the molecular switches that alter the state of compaction of chromatin to allow gene activation or repression [1–3]. Some histone modifications like acetylation and phosphorylation are highly dynamic, whereas the methylation have been regarded as ‘‘permanent’’ chromatin marks.

Methylation of lysine (K) residues at the ε-amino group are one of the most common post-translational modifications of histone N-terminal tails [4, 5]. Lysine residues can be mono-, di- and tri-methylated. Histone lysine methylation plays critical roles in virtually all chromatin-templated biological processes, such as transcription and DNA repair [5, 6]. Cumulative evidence indicates that histone lysine methylation exerts diverse biological functions in a site- and methylation state-dependent manner [5, 6]. Like other histone modifications, histone lysine methylation is reversible and dynamically regulated through the balancing activities of histone lysine methyltransferases and demethylases [7–9]. To date, two families of proteins have been found to possess lysine demethylase activity. Members of one family are amine oxidases that can catalyze demethylation of mono- and di-methylated lysine residues using flavin adenine dinucleotide (FAD) as a cofactor [10]. Members of the other family are the JmjC family proteins that catalyze oxidative demethylation reactions with iron and α-ketoglutarate as cofactors [11–13]. In this review we made a generalized description about the histone lysine demethylases- which includes the two known lysine demethylase families, their role in human tumorigenesis and the potential therapeutic targets of lysine demethylases.

### Historical development of protein methylation

The research on ‘protein methylation’ were first started in the 1960s. In 1962, first Huang and Bonner's [14] investigations stated that the histones might function as gene regulators. Later in 1964 Allfrey *et al.* [15]. observed that the amino acids of histone might be acetylated or methylated. In 1967, Paik *et al.* [16]. and in 1968 Hempel *et al.* [17]. reported the presence of N-dimethyl-lysine in histone hydrolysates in addition to the ε-N-monomethyl-lysine. In 1968 it was the reason for the identification and purification of protein methylase I [18]. The discovery of the arginine methyltransferase was by Baldwin and Carnegie [19], and Brostoff and Eylar [20], independently and they finally reported that Arg107 is methylated at the guanidino group. In 1967 Liss and Edelstein [21] identified another enzyme called protein methylase II, that is capable of esterifying the dicarboxylic amino acid residues of proteins. It was the second enzyme discovered that transfers a methyl group to a protein side chain. In 1970 protein lysine methyltransferase, which methylates the ε-amino group of lysine residues in a protein from calf thymus was characterized and Identified as protein methylase III [22, 23]. Now, these enzymes are known as a family of lysine specific histone methyltransferases [3]. In the early 1980s, it was known that the specific enzymes methyltransferases were responsible for methylation of lysine, arginine, histidine and dicarboxylic amino acids. In the 1990s, a vast research in protein methylation occurred and now it is clear that protein methylation carries many important biological functions, including gene regulation and signal transduction.

The protein methylase I have two subtypes: (i) histone or heterogenous nuclear ribonucleoprotein (hnRNP)-specific and (ii) myelin basic protein (MBP)-specific. In the mid-1990s the knowledge of protein- arginine methylation and its applications were vastly spread. It is now recognized that Protein methyl transferase (PRMT) exists as a family with several subtypes. The PRMT family has been shown to include at least nine methyltransferases, designated as PRMT 1–9 based on differences in primary sequence and substrate specificity [24–28].

### Types of protein-methylation

The protein-methylation was by specific enzymes called protein methyl transferases. These protein methyl transferases can be classified into two major groups: The first group modifies carboxyl groups to form methyl esters. These are reversible reactions and can regulate the activities of the methylatable protein. The second group of protein-methylation reactions is irreversible methyl transfer to sulfur and nitrogen atoms. The function of these reactions is much less clear but appears to be the generation of a variety of new amino acids for specialized cellular roles.

### Glutamate methylation in bacteria

Glutamate methylation was observed in bacteria but not in eukaryotic cells, particularly the chemotactic bacteria glutamate methylation was common but Robert Sprung first time reported glutamate methylation in bacteria [29]. The enzyme recognizes the sequence (S/A)-(S/A)-X-X-(F/Q)-(F/Q)-X-A-A (here X represents a variety of amino acids) and then methylate's a glutamate residue in the sixth position [30, 31]. The enzyme responsible for the demethylation reaction was the CheB methylesterase, can also recognize glutamine residues and catalyzes their deamidation as well as the demethylation of glutamate γ-methyl esters. Both the glutamate methyl ester and glutamine residues are uncharged at neutral pH, while the glutamate residue has a net negative charge. Thus, the methylation/demethylation system can convert receptors with glutamine/γ-glutamate methyl ester residues with one type of signaling function to receptors with glutamate residues and the second type of signaling function [32]. Initially, these bacterial enzymes were the examples of a family of glutamate methyltransferases/methylesterases that could reversibly modify other bacterial and animal cell proteins and regulate their activity. However, glutamate methylation has not found to be utilized in eukaryotic cells, or even in non-chemotactic systems in bacteria.

### Cysteine and leucine methylation

Cysteine and leucine methylation was observed in eukaryotic cells but not in prokaryotic cells. It was observed in carboxyl-terminal of isoprenyl cysteine and leucine residues. This type of methylation may control the assembly and disassembly of nuclear lamins [33, 34]. Evidence has also been presented for a role of this type of methylation in signal transduction reactions involving G proteins. Inhibitor studies have suggested more specific roles of the methylation reaction in platelet stimulation leading to their aggregation [35] and in the response of macrophages [36], neutrophils [37] and HL-60 granulocytes [38] to chemotactic stimulation.

### Histidine methylation

Methylation Of histidine residues has been described in actin, myosin, histones and rhodopsin [39]. The role of actin methylation has been tested by creating actin variants that contain an arginine or tyrosine residue in place of histidine-73 [40]. Several properties of these variants, including their ability to become incorporated into the cytoskeleton of a non-muscle cell, were found to be identical to those of normally methylated actin. However, actin interacts with a very large number of proteins and the conserved methylhistidine residue may be crucial for one or more of these interactions.

### Asparagine methylation

The side-chain amide nitrogen of the asparagine-72 residue of the g-subunit of the C- and R-phycocyanins and the B-, C- and R-phycoerythrins (photosynthetic accessory proteins present in cyanobacteria and red algae) is methylated. On the C-phycocyanin, this methylated residue is located near one of the chromatophoric groups, and evidence has been presented that methylation serves to enhance the efficiency of energy transfer in the light-harvesting complexes [41, 42].

### Arginine methylation

Arginine methylation has been identified down to the earliest branches of eukaryotes [43, 44]. It can be either activatory or repressive for transcription. Methylation of arginine is regulated by arginine methyltransferases (PRMTs), but there are no enzymes yet identified that can reverse arginine methylation [45]. Arginine methylation was initially thought to be a rather permanent modification [46], but there are several analyses supporting a reversible nature of methyl arginine [47]. The estrogen receptor α (ERα) is methylated in an RGG motif at R260 by PRMT1 [48]. Methylation of arginine is catalyzed by a group of nine protein arginine methyltransferases [49]. The PRMT family of enzymes is well conserved within multicellular organisms ranging from cnidarians to humans [50]. Methylated arginine residues have been found in both histone and non-histone proteins as myelin basic protein, myosin, heat shock proteins, nuclear proteins, ribosomal proteins, and tooth matrix proteins. These methylated arginine residues involved in functions such as RNA processing, DNA repair, and transcription [51]. In histones, the best-characterized methylated-arginine residues include R2, R8, R17, and R26 of histone H3 and R3 in histone H4 and H2A [52]. Due to the methylation state of individual arginine residues, the adjacent chromatin region is either transcriptional active or repressed. Asymmetric di-methylation of H3R2, for instance, has been linked to inactive chromatin regions. In contrast, mono-methylation or symmetric di-methylation of H3R2 is associated with an active chromatin state [53, 54].

### Lysine methylation

The discovery of LSD1, another protein family with more than 30 histone demethylases structurally different from LSD1 was described, all of which share a motif designated the Jumonji C (JmjC) domain and revealing substrate specificity. Identification of these enzymes opened a new era in understanding how chromatin dynamic is regulated and further understanding of the regulation of these enzymes will provide significant insight into fundamental mechanisms of many biological processes and human diseases. Six lysine (K) residues on histone H3 and H4 (H3K4, H3K9, H3K27, H3K36, H3K79 and H4K20) are subjected to mono, di and tri methylation. Importantly each methylation state represents a specific epigenetic mark with a precise biological meaning and well defined chromatin localization [2]. H3K4, H3K36, and H3K79 are implicated in activation of transcription, whereas H3K9, H3K27 and H4K20 are connected to transcriptional repression.

### Histone lysine demethylation

Histones may be methylated on either lysine (K) or arginine (R) residues. Methylation of histone does not affect the chromatin structure but they create a binding sites for regulatory proteins [55]. Arginine side chains may be mono or di-methylated as symmetrically or asymmetrical, whereas the lysine side chains can be mono, di, or tri-methylated. As there are number of distinct lysine and arginine residues present in the N-terminal tails of histones the methylation possibilities were more at this sites. Lysine methylation was limited to certain proteins that recognize the modifications. For instance, hetero chromatin protein HP1 binds to H3k9 where as chromodomain helicase DNA binding protein CHD1 recognizes H3K4 [2]. On the other side p53-binding protein 1 (p53BP1) recognizes methylated H3K79 [56], and the WDR5 a vertebrate transcriptional activator recognize both di and tri methylated H3K79 [57]. All methylated residues play a key role in transcription and DNA damage [57]. Methylation of lysine is by histone methyltransferases (HMTs) and as there are two classes of lysine methyl transferases have been identified, the proteins with amine oxidases LSD1 and LSD2. Both LSD1 and LSD2 use FAD-cofactor and releases demethylated lysine, FADH_2_ and formaldehyde. The JmjC-domain, another class of lysine demethylases uses iron and α-ketoglutarate as cofactors leads to release succinate, carbon dioxide, and formaldehyde along with demethylated lysine. The histone lysine methylation regulation landscape has been changed considerably in the recent years but the biological functions of lysine demethylases are uncovered. Lysine methylation was associated with different types of cancers. The mutations or overexpression of several lysine demethylases associated with cancer has been raising the possibility of therapeutic potential. Past evidence suggests that HDMs participate in nuclear receptor [58]. For instance the H3K4-Me associated with prostate cancer, H3K4-Me2 was associated with lung, kidney, prostate and breast cancers, H3k9-Me2 with gastro adenocarcinoma and H3K27-Me3 with breast and ovarian cancer [55]. Transcriptional activation by nuclear receptor is a multistep process and involves primary and secondary coactivators. The binding of hormone to the receptor induces a structural reorganization which allows the nuclear receptor to recognize specific DNA elements at promoter regions [59]. These changes will result in recruitment of coactivators, leads to intrinsic enzymatic activities, such as demethylation. At the same time these proteins may change the local chromatin structure and subsequent recruitment of RNA polymerase II transcriptional machinery [60]. The histone demethylases were the key players in the regulation of androgen receptor (AR) -mediated transcription. Both LSD1 and KDM4C interact with AR and induce demethylation of H3K9 at the prostate-specific antigen (PSA) promoter [61, 62]. Similarly, transcriptional activation of thyroid receptor leads to demethylation of H3K9 by a JmjC-domain containing family member [63]. Although studies associated with arginine methylation to estrogen receptor (ER) function, very little has been done in elucidating the role of lysine methylation in regulating ER-dependent transcription. Several H3K9 histone methyl transferases have been involved in repressing ER targets pS2 and GREB1 in the absence of estrogen [64]. List of targets that corresponds to the methylation/demethylation sites and their role in transcription was tabulated in Table 1.

**Table 1:** List of targets that corresponds to the methylation/demethylation sites and their role in transcription

Methylation site to be modified by KMT/KDM	Targets	Role in transcription
H3k4	MLL, ALL1,ALR1, ASH1, Set 1, Set 9/7, NSD1	Activation
H3K9	SUV39H, G9a, EU-HMTase/GLP, ESET/SETBD1, CLL^*^, Spclr4	Activation/Repression
H3K27	EZH2	Repression
H3K36	HYPB, Symd2, NSD1, Set2	Elongation
H3K79	Dot1	Activation
H3K20	PrSet7/8, SUV420H, SpSet9	Silencing

### Structure of histone lysine demethylases

Histone lysine demethylases are most frequent targets for epigenetic drugs and are gaining more interest in research. A lysine in histone proteins can be mono-, di-, and trimethylated. Every modification on the each/same amino acid can have different biological effects [65]. The recent studies on histone lysine demethylases reveal two types of enzyme mechanisms [66]. The iron-dependent enzymes, which can demethylate side chains of lysine in all three methylation states. With reference to this, the oxidative chemistry that involves in the function of flavin-dependent histone demethylases makes it impossible for these enzymes to act on trimethylated lysine and restricts their activity to mono- and dimethylated substrates [67]. The two main flavoenzyme demethylases are LSD1 and LSD2 also known as KDM1A and KDM1B [10, 68].

### LSD1

The first FAD-dependent histone lysine demethylase family identified was LSD1 [10] and its homolog LSD2. The LSD1 structure consists of three domains, the amine oxidase domain, FAD binding domain and SWIRM domain. The FAD dependent amine oxidase domain consists of an active site to bind substrates. The FAD binding domain consists of a tower domain which interacts with CoREST and the SWIRM domain function in LSD1 was unknown but the SWIRM domain in other histone protein modifications binds to DNA as it is having solvent exposed positive charged patch. The tower domain has an insert of 102 amino acids which seperates N and C terimus of amine oxidase domain. The tower domian is linked with the corepressor protein CoREST is essential for demethylation and removes methyl groups from mono- and dimethyl Lys4 of histone H3, a gene activation mark [69]. These enzymes are often deregulated in human diseases, it is essential to understand their physiological and pathological/biological functions by elucidating their exact structure, regulation and substrate specificity. The enzyme is an attractive target for epigenetic drugs as it is overexpressed in solid tumors [70], its involvement in various infection, differentiation processes [71, 72].

### LSD2

**Figure 1:**
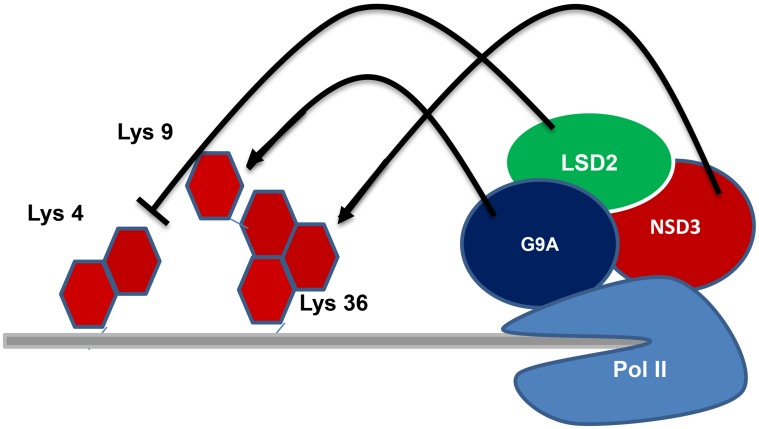
Regulation of transcriptional elongation: LSD2 associated with transcriptional elongation factors and phosphorylated RNA polymerase II

LSD2 is also like LSD1 which has specificity in removing methyl groups from mono and dimethyl Lys4 of H3. But, the biology of LSD2 is different from that of LSD1 as LSD2 does not bind CoREST [73, 74]. The catalytic domains of these LSD1 and LSD2 proteins shows 45% identity and is structurally homologous with the amine oxidases that act on biogenic amines [10, 67]. Among all these proteins, a vast research for more than 50 years was on human monoamine oxidases (MAOs) A and B which led to the development of a multitude of inhibitors including antidepressive and antiparkinson drugs [75]. The LSD1 has been vastly studied [10, 76] when compared to LSD2, the enzyme activity and biological function of LSD2 have become more prominent. Several studies [68, 77] reported that LSD2 particularly demethylates mono- and di-methylated H3K4, but van Essen *et al.* [78] reported that LSD2 also demethylates dimethylated H3K9. The LSD2 requires a long stretch of H3 tail sequence for demethylation of H3K4 [68]. Biochemical studies of LSD2 indicate that it is associated with transcriptional elongation factors and phosphorylated RNA polymerase II [77, 79] (Figure 1). LSD2 is enriched in the coding regions of actively transcribed genes and has a role in maintaining a repressive chromatin environment within gene bodies through its H3K4 demethylase activity [77]. The H3K4 demethylation by LSD2 indicates that it is linked with the establishment of maternal imprinting in mouse oocytes [73]. As in LSD1 the LSD2 also contains a N-terminal SWIRM domain and a C-terminal amine oxidase domain. But the LSD2 does not have tower domain which is a particular insert present in the amine oxidase domain of LSD1 that is required for LSD1 binding of CoREST and LSD1's enzymatic activity [69, 80, 81]. The LSD2 has a N-terminal region that is absent in LSD1, which forms a CW-type zinc-finger domain [74]. The CW domain is present in several chromatin-remodeling proteins and has capable to bind methylated histones [82, 83]. Deletion mutant experiments explained that the N-terminal zinc finger domain and the SWIRM domain are essential for LSD2 demethylase activity [74].

### Crystal structure of LSD1

Crystal structure of LSD1 represents a number of transcriptional corepressor complexes like CoREST, CtBP, and HDAC complexes which plays a major role in silencing neuronal-specific genes in nonneuronal cells [84–89]. The C-terminal FAD dependent amine oxidase with a tower insert occupies major part in the LSD1 structure [90–92]. The N-terminal of LSD1 contains SWIRM domain, which was often found in chromatin remodeling and modifying complexes with unknown function [93]. The SWIRM and FAD dependent amine oxidase domains pack together through extensive forces and form a globular structure. The SWIRM domain contains mostly the α-helices (six-helical bundle structures characterized as a long helix and in the center it was surrounded by five other helices) and the amine oxidase domain exhibits topology with several Flavin dependent oxidases. In addition to this the SWIRM domain has two stranded β sheets at the C-terminal end which helps to anchor the interactions between the SWIRM and AOL domains. The amine oxidase domain has two sub domains, a FAD binding sub-domain and a substrate binding subdomain. The FAD binding sub-domain has a mixed structures with both α-helices and β-sheets. The substrate binding domain has six stranded mixed β sheet flanked by six α helices. The tower insert of amine oxidase domain consists of a pair of long helices that adopts a typical antiparallel coiled confirmation [94].

Demethylation of mono or di-methylated lysine on histone is catalyzed by LSD1. This enzyme has an amine oxidase containing flavin adenine dinucleotide (FAD) as an electron acceptor to first oxidize the lysine N-methyl amine to lysine N-methylimine. FADH_2_ is re-oxidized to FAD by molecular oxygen producing hydrogen peroxide. The N-methylimine is non-enzymatically hydrolyzed to a carbinolamine which spontaneously dissociates to a demethylated lysine and formaldehyde. If the substrate is a dimethylated lysine the enzyme performs sequential removal of both methyl groups by the same mechanism. LSD1 demethylates histone H3 as part of a multimeric protein complex in which it directly interacts with the co-repressor of the repressor element 1 silencing transcription factor, CoREST. Interaction of these two proteins has been studied in detail and the crystal structure of the complex containing an analog of the histone H3 peptide was determined and reported [81, 95].

### LSD1 mechanism and function

**Figure 2:**
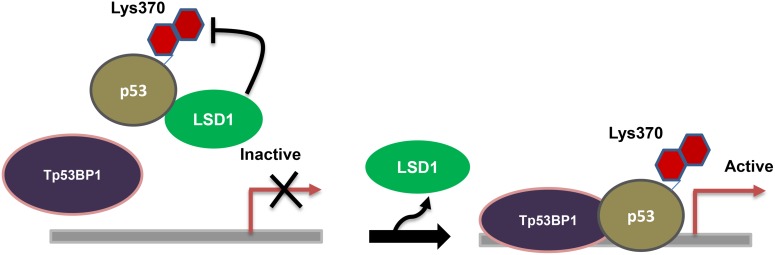
Regulation of p53: LSD1 demethylate lysine 370 of the tumor suppressor p53, repressing its function by interacting with 53BP1

As LSD1 uses FAD as cofactor, it is a member of the FAD-dependent amine oxidase class lysine methylases. Demethylation by LSD1 occurs by oxidizing the carbon-nitrogen bond between the methyl group of lysine residues, and produce demethylated lysine, FADH_2_ and formaldehyde. LSD1 can only demethylate mono- and dimethylated lysines, but not trimethylated lysines, as they need a lone pair of electrons on the amine group to from imine. Initially LSD1 was reported as gene repressor as it demethylate H3K4 and repress the transcription of neuron [85], but the LSD1 was also able to activate the gene as it demethylate H3K9 which leads to upregulate the expression of gene [61]. Garcia Barcet etal reported that 80% of the promoters occupied by LSD1 were bound to RNA polymerase II, suggesting that LSD1 was liked more in active genes rather than the inactive genes [64]. LSD1 has been involved in the maintenance of diseases such as neuroblastoma [70] and high-risk prostate cancer [96]. This enzyme plays important regulatory roles in cell differentiation [97] and it is also required for transcriptional repression of the hTERT gene in both normal and cancerous cells [98]. Other than histone substrates, LSD1 was shown to demethylate K370 of the tumor suppressor p53, repressing its function by interacting with 53BP1 (Figure 2), a DNA damage checkpoint [99]. The SNAG domain of Snail1 seems a H3-like structure and functions as a molecular hook for recruitment of LSD1 to repress gene expression in metastasis [100]. Further, LSD1 is highly expressed in breast cancer cells and it is an essential mediator of the inter chromosomal interactions necessary for estrogen-dependent transcription [101, 102]. Downstream target genes regulated by lysine-specifc demethylase 1 (LSD1) in colon cancer cells was investigated by Jiang Chen [103]. They report that total 3633 and 4642 differentially expressed genes were signifcantly upregulated and downregulated respectively in LSD1-silenced SW620 cells.

### Biochemical characteristics of lysine methylation

The protein lysine methyl transferases and protein lysine demethyal transferases have a key role in epigenetic regulation. The protein lysine methyl transferase methylates histone H3 at lysine 27 (polycomb repressive complex) is over expressed in different types of cancer [104]. Polycomb-repressive complexes has important role in controlling the expression of downstream genes and it also promotes cell cycle progression in physiological and pathological conditions [105]. Similar reports on protein lysine methyl transferases and protein lysine demethyl transferases promote malignant transformation via histone methylation dependent transcriptional regulation [106–110].

### Lysine methyltransferases

Mono, di and trimethylation of the lysine ε-amino group are catalysed by lysine methyltransferases in an S-adenosyl methionine dependent manner [111]. Histone methyl transferases (HMT) consists of two main classes, the SET domain family and DOT1 family. Most lysine methyltransferases contain the SET domain which has N-methyl transferase activity, but some non-SET-domain proteins like DOT1L methyltransferase like 10 (METTL10) and METTL21A are also known to have lysine N-methyltransferase activity [112, 113]. DOT1 methyl transferases lacks SET domain and methylate lysine 79 with in the core domain of H3. DOT1 methyl transferases can only methylate nucleosomal substrates but not free histones. The SET-domain methyl transferases catalyze a sequential bi-bi kinetic mechanism in which both substrate association and product release occur in a random manner [111]. The methylation increases the hydrophobic and basic nature of the lysine residue, which allows other proteins to recognize methylated lysine.

### Lysine demethylases

The methylation of lysine residues is by lysine demethylases and this is an irreversible reaction. Lysine demethylases are a group of proteins that are categorized into two functional enzymatic families. The first family includes LSD1 and LSD. LSD1 is a flavin-dependent monoamine oxidase and the LSD2 is the only homologue of LSD1 in the human genome [77]. These amine oxidases can only demethylate mono and dimethyl lysine residues, but not trimethyl lysine residues because they require a lone pair of electrons that are not present on trimethyl lysine residues. The second family of lysine demethylases is JmjC domain containing proteins which are metalloenzymes that can use an oxygenase mechanism to demethylate mono, dimethyl and trimethyl lysine residues.

### Roles of KMTs and KDMs in human tumorigenesis

Lysine methylases and demethylases are the factors for development of many diseases, especially cancer. LSD1 is over expressed in various types of cancer like bladder and colorectal cancer, prostate cancer and oestrogen-receptor-negative breast cancer [114, 115]. Over expression of LSD1 was found to prevent the differentiation process and hold the malignant phenotype in neuroblastoma. The p53 activity was decreased when LSD1 was directly interacted with p53, which is associated with tumorigenesis [99]. In addition, lysine demethylases was not found to be altered in many cancers though it may functions as ubiquitin ligase. In the other side recurrent mutations in genes that encodes histone demethylases have been discovered in the gene encoding ubiquitously transcribed TPR protein on the X chromosome, where inactivating mutations are present in various types of tumors [116, 117], indicating the role of lysine methylases in human tumorigenesis. KDM6A catalyses the demethylation of H3K27me2 and H3K27me3, and a mutation in KDM6A is therefore to lead to an increase in H3K27 methylation, which is functionally equivalent to the enhanced EZH2 activity found in different human tumours [118, 119]. Recent studies on LSD1 activity have therapeutic potential in cancer, which includes the finding of LSD1 that is necessary for the maintenance of acute myeloid leukemia containing mixed lineage leukemia translocations [120] and inhibition of LSD1 can reactivate the all-trans-retinoic acid differentiation pathway in acute myeloid leukemia [121]. In addition to providing genetic evidence for a role of LSD1 in a mouse model of mixed lineage leukemia AF9 induced leukemia, Harris *et al.* [120]. showed that the MAO inhibitor tranylcypromine significantly inhibited the colony-forming ability of AML cells.

The JMJD2 subfamily member's demethylases H3K9me3 and H3K9me2 as well as H3K36me3 and H3K36me2 which are overexpressed in cancer. The locus containing the JMJD2C (GASC1) gene is genomically amplified in squamous cell carcinoma, medulloblastoma and breast cancer [122, 123]. The exhaustion of JMJD2B slows the growth of various cancer cell lines [124–127]. The development of cancer in different mouse models was linked with the loss of H3K9 trimethylation. Here, loss of the two specific H3K9me3 histone methyltransferases SUV39H1 and SUV39H2 in double-knockout mice results in genomic instability and the growth of cancer [128]. The decrease in H3K9me3 levels can also increase carcinogenesis in a T cell lymphoma mouse model [129]. The over expression of H3K9me3 and H3K9me2 by JMJD2 demethylases would show same effects, and targets the enzyme activity of the JMJD2 subgroup of Jumonji proteins could therefore have therapeutic potential. as discussed above, proof of concept has been achieved for JMJD2B, and different studies have also shown that JMJD2C is required for growth of various cancer cell lines like prostate carcinoma [62], breast carcinoma [122], squamous cell carcinoma [130] and diffuse large B cell lymphoma [131]. The H3K4me3 and H3K4me2 demethylase by JARID1B binds to H3K4me3-enriched promoters and it is required for normal development [132, 133]. This protein is overexpressed in various cancers like prostate cancer, breast cancer and bladder cancer [134, 135]. It was reported that JARID1B is necessary for the growth of a breast cancer cell line [136] and for the continuous growth of melanoma [137].

In addition to JMJC domain a multifunctional protein KDM2B also catalyzes the demethylation of H3K36me2 and H3K36me1 [138]. The catalytic demethylase activity of KDM2B is required for the proliferation of acute myeloid leukemia and pancreatic ductal adenocarcinoma, and it also found to be overexpressed [139, 140]. The previous research explains that KDM2B regulates the expression of polycomb target genes [138, 141] suggesting that KDM2B might contribute to tumorigenesis through the regulation of these genes.

### Functions of protein lysine demethylases

Protein lysine methylation appears to play a key role in gene repression, gene activation and in gene differentiation process. Several posttranslational modifications of histones on specific amino acids like altering chromatin structure or by recruiting enzyme regulatory complexes or the combined effects determine the outcome of transcription, either activating or suppressing gene expression [45, 90]. The general functions of protein lysine demethylases in are explained below.

### Gene repression

The LSD1-CoREST-HDAC core is involved in various biological process like in like hematopoiesis where it interacts with growth factor independent1 transcription repressor and repress the target genes. It also involved in silencing mature B-cell genes [142] by interacting directly with the transcriptional repressor B lymphocyte-induced maturation protein-1 (Blimp-1). This LSD1-CoREST-HDAC also interacts with constitutive transrepressor TLX and forms a complex to repress PTEN gene and inhibiting cell proliferation [143]. LSD1 can also interact directly with p53 to show p53-mediated transcriptional repression and repress the alpha-fetoprotein. The well-known p53 target gene p21 can transcribe actively without LSD1.

### Gene activation

**Figure 3:**
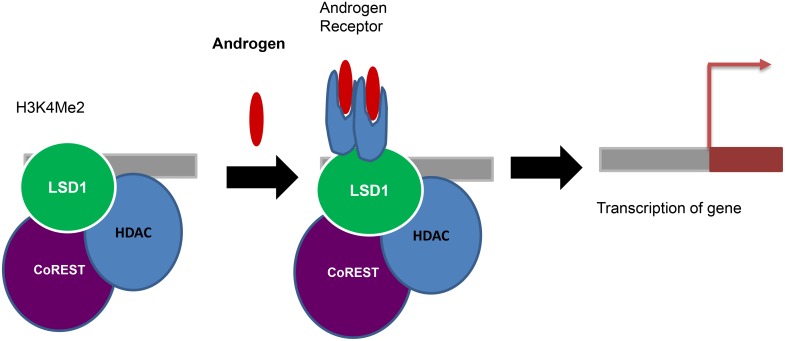
Coactivator function: Androgen and LSD1 colocalize on promoters and stimulate H3K9 demethylation without altering the H3K4 methylation status and promote ligand dependent transcription of AR target genes resulting in enhanced tumor cell growth

The activation of androgen and estrogen receptors requires LSD1 dependent histone H3K9 demethylation [61, 64]. AR and LSD1 colocalize on promoters and stimulate H3K9 demethylation without altering the H3K4 methylation status and promote ligand dependent transcription of AR target genes resulting in enhanced tumor cell growth (Figure 3). LSD1 knock-down decrease the activation of AR-responsive promoters. Genome-wide analysis of LSD1 promoter occupancy following estrogen treatment of MCF7 cells has showed better results regarding the activatory role of LSD1 [64]. The activation markers like H3K4-me2 and acetyl-H3K9 suggesting that LSD1 is involved in gene activation rather than repression. The two roles of LSD1 in gene repression and activation is also explained by the fine regulation of growth hormone expression during pituitary development [144].

### Development and differentation

The functional role of LSD1 has been actively investigated and it appears to be key in development and differentiation. Knockout of LSD1results in mouse embryonic lethality at or before embryonic day 5.5 [97, 145]. The zygotic LSD1 expression initially appeared at the morular stage and became wide in postimplantation embryos. ES cells derived from LSD1 knockdown mouse showed severe growth impairment, it may be due to increased cell death, impaired cell cycle progression, and defects in differentiation. Conditional knockout of LSD1 showed defects in pituitary gland development and the Notch signalling pathway [97]. The interference RNA inhibition of LSD1 in several mammalian haematopoietic lineages resulted in impairment of differentiation *in vitro* [146].

### Functions of lysine methylation

#### Effect on other protein modifications

According to the previous reports the protein lysine methylation affects other post translational modifications by directly through competitive inhibition or indirectly. The lysine methylase EZH2 methylates histone H2B at lysine 120, which is a site of ubiquitylation, inhibits ubiquitylation and suppresses the transcription of genes involved in cancer [147]. Besides to this, lysine methylation also affects phosphorylation and acetylation of neighbouring distinct amino acids. So, methylated lysine residues change the affinity of enzymes such as kinases or phosphatases for their substrates, which may alter post translational modifications at other sites on the other substrates [148, 149].

### Interacting protein partners

**Figure 4:**
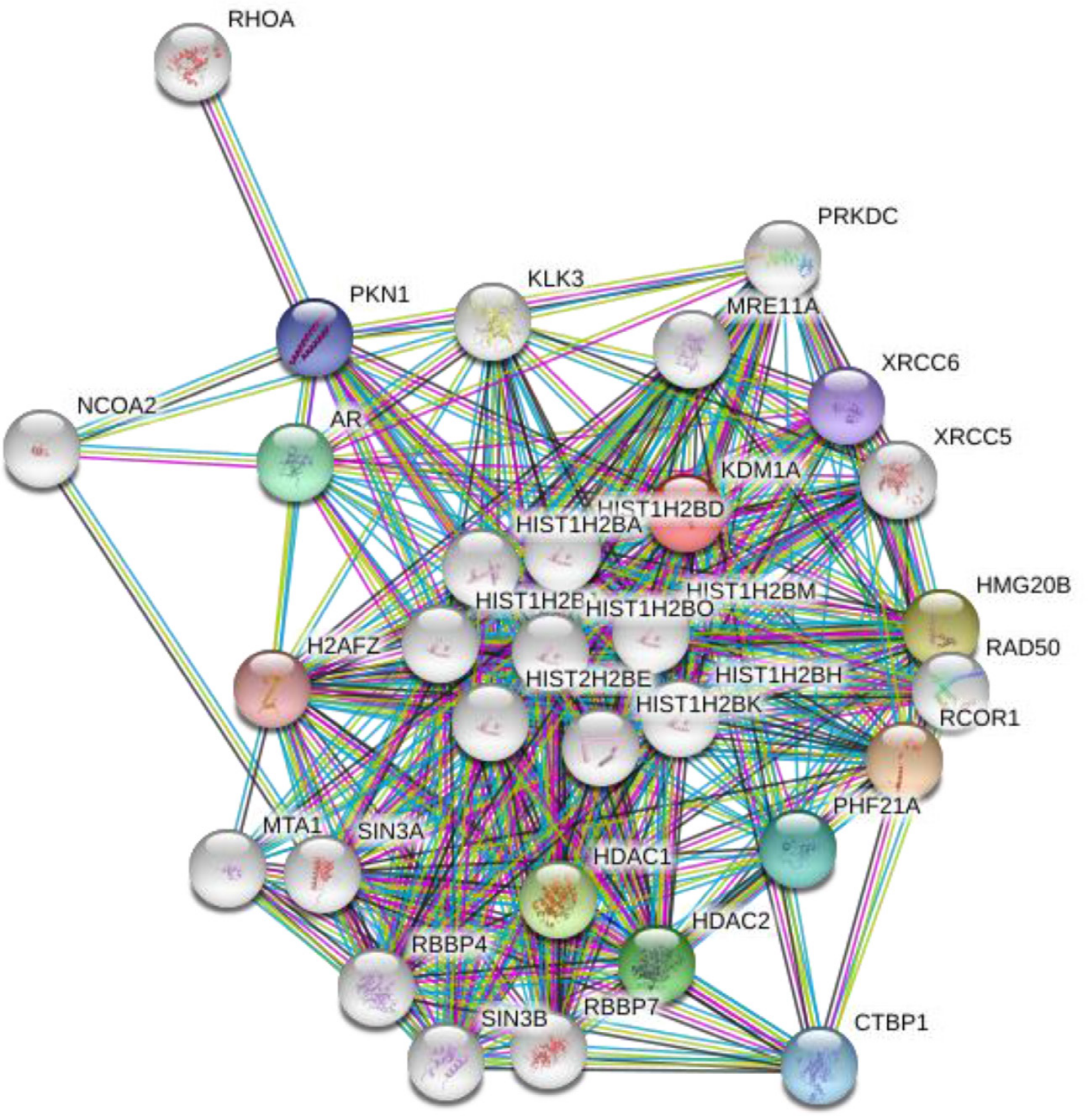
Interacting proteins map for LSD1: A group of proteins that interact with LSD1, picture was generated by STRING software

Lysine methylation can regulate interactions with other proteins. Biochemically, methylation of lysine residues doesn't change or neutralize its charge and has a small change in size, but it changes the hydration energies and hydrogen bonding potential of side chains in lysine residues. There are some methyl lysine binding proteins which have a specific motif like chromodomain for recognizing the methylation states of lysine, called the ‘aromatic cage’ which contains a collection of aromatic proteins, often accompanied by one or more neighbouring anionic residues [150]. The combination of favorable cation-π, electrostatic, and van der Waals interactions, as well as size matching, gives these proteins a high degree of specificity for the methylation state [151]. By contrast, the PP2A phosphatase complex (serine/threonine protein phosphatase 2A), a key negative regulator of the MAP Kinase pathway, binds to MAP3K2 and this interaction is blocked by methylation, implying that methyl lysine can also block protein-protein interactions [149]. All the protein interactions can influence the functions lysine demethylases and its involvement in DNA methylation. LSD1 interacting partners and their functions are represented in the Table 2. A picture was also generated by STRING software (Figure 4).

**Table 2:** List of interacting proteins with lysine specific demethylases with their molecular weights and related pathways

KDM1A interacting protein name	Molecular weight (Kda)	Function/related pathway
MTA1	81	Activated PKN1 stimulates transcription of AR (androgen receptor) regulated genes KLK2 and KLK3 and Validated targets of C-MYC transcriptional activation.
RBBP7	47.8	Activated PKN1 stimulates transcription of AR (androgen receptor) regulated genes KLK2 and KLK3 and Cellular Senescence
RBBP4	47.6	
CTBP1	49	Notch signaling pathway (KEGG) and Signaling by Wnt.
HDAC1	55	Activated PKN1 stimulates transcription of AR (androgen receptor) regulated genes KLK2 and KLK3 and PEDF Induced Signaling
HDAC2	55	
XRCC5	82	DNA Double-Strand Break Repair and DNA damage_NHEJ mechanisms of DSBs repair
XRCC6	69.8	
PRKDC	461	
RHOA	22	Bisphosphonate Pathway, Pharmacodynamics and Development VEGF signaling via VEGFR2 - generic cascades.
SIN3B	133	PEDF Induced Signaling and Development NOTCH1-mediated pathway for NF-KB activity modulation
KLK3	28.7	Adhesion and Transcription Androgen Receptor nuclear signaling.
HIST2H2BE	14.2	DNA Double-Strand Break Repair and Activated PKN1 stimulates transcription of AR (androgen receptor) regulated genes KLK2 and KLK3.
HIST1H2BK	13.8	
HIST1H2BJ	13.9	
HMG20B	33.9	Activated PKN1 stimulates transcription of AR (androgen receptor) regulated genes KLK2 and KLK3 and Factors involved in megakaryocyte development and platelet production.
NCOA2	159	CCR5 Pathway in Macrophages and Circadian rythm related genes.
PHF21A	74.8	Factors involved in megakaryocyte development and platelet production and Response to elevated platelet cytosolic Ca2+.
AR	110	CCR5 Pathway in Macrophages and Transcription_Role of VDR in regulation of genes involved in osteoporosis.
RCOR1	74.8	fMLP Pathway and Respiratory electron transport, ATP synthesis by chemiosmotic coupling, and heat production by uncoupling proteins.
PKN1	103.9	Signaling mediated by p38-gamma and p38-delta and Translation Translation regulation by Alpha-1 adrenergic receptors.
PRKDC	469	DNA Double-Strand Break Repair and DNA damage_NHEJ mechanisms of DSBs repair.
RAD50	146	
MRE11A	81	DNA Double-Strand Break Repair and Resolution of D-loop Structures through Synthesis-Dependent Strand Annealing (SDSA).
KDM1B interacting proteins	Molecular weight (Kda)	Function/Related Pathway
ASXL1	165.4	Deubiquitination and Metabolism of proteins
ASXL2	98.3	
MBD5	160	
MBD6	101	
FOXk2	78	
HCFC1	208	Mitochondrial Gene Expression
BAP1	91	DNA Double-Strand Break Repair and Deubiquitination.
HIST2H2AA3	14	Activated PKN1 stimulates transcription of AR (androgen receptor) regulated genes KLK2 and KLK3 and Meiosis.
HIST2H2AC	14	
HIST2H3A	16.9	
HIST3H2BB	13.9	DNA Double-Strand Break Repair and Activated PKN1 stimulates transcription of AR (androgen receptor) regulated genes KLK2 and KLK3.
HIST1H2BA	14.16	
HIST1H2BN	13.9	
HIST2H2BF	13.9	
HIST2H2BE	13.9	
HIST1H2BD	13.9	
HIST1H2BB	13.8	
HIST1H2BO	13.9	
HIST1H2BK	13.9	
HIST1H2BJ	13.9	

### Protein stability

The lysine methylation block the action of ubiquitin ligase, preventing proteins from degradation via the ubiquitin/proteasome system and hence the lysine methylation may increase the stability of proteins by preventing polyubiquitylation. In *Saccharomyces cerevisiae*, lysine methylated proteins show a significant longer half-life than proteins which no methylation was found. Furthermore 43% of methylated lysine sites were predicted to be amenable to ubiquitylation, suggesting that methylated lysine residues might block the action of ubiquitin ligase [152]. However, methylation-dependent ubiquitination is carried out by damage-specific DNA binding protein 1 (DDB1)/cullin4 (CUL4) E3 ubiquitin ligase complex and a DDB1-CUL4-associated factor 1 (DCAF1) adaptor, which recognizes monomethylated lysine residues and promotes polyubiquitylation of the other lysine residues on substrates such as retinoic acid related orphan nuclear receptor *α (RORα)* [153], stating that lysine methylation may also destabilize target proteins through regulation of distant polyubiquitylation.

### Subcellular localization

A nuclear localization signal comprises of some short sequences of positively charged lysine or arginine residues exposed on the protein surface [154]. As both lysine and arginine residues are critical for the nuclear localization of proteins, one could mediate that methylation of lysine or arginine residues may influence subcellular localization. Infact, some lysine methylated proteins like heat shock protein 70 and p53 are commonly localized in the nucleus, whereas the unmodified versions of these same proteins are localized in both the cytoplasm and the nucleus [155, 156].

### Promoter binding

The methylation of lysine regulates the binding affinity of transcription factors for promoters, that changes the transcription levels of target genes [157]. For instance, lysine methylation of p53 by the SETD7 and the nuclear factor κB by the methyl transferase nuclear receptor-binding SET domain-containing protein 1 distinctly increase their ability to bind the promoter and activation of downstream genes [156, 158]. In a structural modelling analysis of RELA(a subunit of NF-κB) with DNA complex, two methylated lysine's on RELA were interact with DNA through hydrophobic contacts [159]. This report indicates that increased hydrophobicity of lysine residues by methylation can increase the promoter binding ability of transcription factors.

### Lysine methylation as a therapeutic target

It is clear that the existing evidence showed that the inhibition of lysine methylase is key role for the development of therapeutic agents. The clinical use of lysine methylase inhibitors gives a better explanation of antitumor effects particularly in animal models of cancer. The fungal metabolite Chaetocin, the first described lysine methylase inhibitor was specific for the methyltransferase SU(VAR)3-9 both *in vitro* and *in vivo* and therefore it was used to study heterochromatin-mediated gene repression [160]. Later, in 2011 Daigle *et al.* [161]. reported the clear evidence of PKMT inhibition by DOT1L inhibitor could lead to selective cancer cell killing and resultant *in-vivo* efficacy in an animal model of cancer. After that, Jo *et al.* [162]. showed that DOT1L has a critical role in hematopoiesis using a postnatal conditional knockout technique. Another inhibitor BIX-01294, which is an inhibitor of the PKMT EHMT2 [163], was shown to effectively suppress the growth of cancer cell lines [108]. Later two anticancer drugs were reported that target EZH2, both inhibit EZH2 carrying a cancer-specific mutation. The first one is GSK126 that targets Y641 and A677 mutated EZH2 for the treatment of DLBCL [164]. GSK126 is a potent, highly selective, S-adenosyl-methionine-competitive, small-molecule inhibitor of EZH2 methyltransferase activity, which effectively inhibits the proliferation of EZH2 mutant DLBCL cell lines and markedly inhibits the growth of EZH2 mutant DLBCL xenografts in mice [164]. The second anticancer drug is EPZ005687, it also targets Y641 and A677 mutated EZH2 found in nonHodgkin lymphoma where it reduces H3K27 methylation in lymphoma cells [165]. Recently, Knutson, S. K. *et al.* [166]. reported that the selective inhibition of EZH2 by EPZ-6438 leads to potent antitumor activity in EZH2 mutant non-Hodgkin lymphoma. EPZ5676, a potent and selective aminonucleoside inhibitor of the PKMT DOT1L [167, 168], has also been studied for the treatment of patients with acute leukaemia in which the MLLgene has undergone rearrangement or tandem duplication.

### LSD1 inhibitors

As the importance of LSD function and their role in different diseases was widely spreaded the recent research was focused on LSD inhibitiors. The inhibitors targeting LSD1 have been actively studied for biological and biochemical charaterizations which help for drug development. Initially the catalytic domains of the LSD show homology with MAOA and MAOB. Some MAO inhibitors including tranylcypromine and phenelzine were found to inhibit nucleosomal demethylation of H3K4 [169]. The phenelzine was more potent against LSD1, whereas the tranylcypromine acts as an inhibitor in cellular environments [169, 170]. The tranylcypromine inhibitor forms a covalent adduct with FAD factor in amine oxidase domain [171]. The wide spread knowledge in H3K4 methylation were observed in embryonal carcinoma cells after treatment with tranylcypromine [169]. Thus the derivatives of tranylcypromine with enhanced potency and target selectivity for LSD were identified and shown to induce differentiation of promyelocytic leukemia cells and slow the growth of prostate cancer cell lines [170, 172]. Pargyline a clinicially useful molecule inhibits MAO B, has also been reported to inhibit LSD1 at H3K9 in the presence of the androgen receptor [61]. The propargylamine or aziridine derived from pargyline was proposed to inhibit LSD1 activity [173]. The aziridine peptide exhibited as a reversible competitive inhibitor, but the propargylamine peptide functions as time-dependent inactivation of the LSD1 by modification of the FAD. Other polyamine derivatives includes bisguanidine and biguanide have been shown to be effective noncompetitive LSD1 inhibitors and treatment results in re-expression of silenced tumor suppressor genes in colon cancer [174, 175]. Recently these polyamine derivatives have been shown to modulate gene expression in breast cancer cells [176].

### JmjC demethylases inhibitors

In addition to the LSD family proteins, JmjC domain containing proteins also have lysine demethylase activity. JMJC demethylases share some similarities with HDACs, and their substrates are also present a lysine side chain with its N-terminus in the proximity of a divalent metal ion [177]. HDAC inhibitors are poor inhibitors of the JMJD2 family of demethylases but few have sufficient potency and target selectivity for LSD were selected and considered for drug development [178]. Kruidenier *et al.* [179]. reported a selective JmjC H3K27 demethylase inhibitor which reduces lipopolysaccharide-induced proinflammatory cytokine production by human primary macrophages. The inhibition of JmjC demethylase proteins may have broad therapeutic application [179], several JmjC containing demethylase have been reported as candidates for anticancer therapy. Inhibitors targeting the Jumonji type demethylase activity showed antitumor effects even though no drugs have yet been evaluated in a clinical trial [180]. Some potential lead compounds specifically GSK-J1 is an inhibitor of the JMJD3 subfamily [179].

## CONCLUSIONS

The present review was focused on the histone lysine demethylases and their role in carcinogenesis. In the recent years, research has been focused in the involvement of histone lysine demethylases in epigenetic regulation of cell differentiation and in tumor growth, specifically for the LSD1 and Jmjc domain containing families. The structure and functions for these families were finely studied to display the enzyme catalytic mechanisms and to develop the drug targets for the tumor growth. But the role/functions of interacting proteins in tumor development of these families mechanisms are unclear. So, the future research has to focus on new manner to find the exact mechanism of LSD when it interacts with other proteins. It's an alternative way to develop new therapeutics. It is also suggested that the finding of new histone-regulating enzymes related to histone demethylases will be well beneficial to the field.

## References

[1] Fischle W, Wang Y, Allis CD (2003). Histone and chromatin cross-talk. Curr Opin Cell Biol.

[2] Margueron R, Trojer P, Reinberg D (2005). The key to development: interpreting the histone code?. Curr Opin Genet Dev.

[3] Martin C, Zhang Y (2005). The diverse functions of histone lysine methylation. Nat Rev Mol Cell Biol.

[4] Barski A, Cuddapah S, Cui K, Roh TY, Schones DE, Wang Z, Wei G, Chepelev I, Zhao K (2007). High-resolution profiling of histone methylations in the human genome. Cell.

[5] Zhang Y, Reinberg D (2001). Transcription regulation by histone methylation: interplay between different covalent modifications of the core histone tails. Genes Dev.

[6] Lan F, Shi Y (2009). Epigenetic regulation: methylation of histone and non-histone proteins. Sci China C Life Sci.

[7] Allis CD, Berger SL, Cote J, Dent S, Jenuwien T, Kouzarides T, Pillus L, Reinberg D, Shi Y, Shiekhattar R, Shilatifard A, Workman J, Zhang Y (2007). New nomenclature for chromatin-modifying enzymes. Cell.

[8] Jenuwein T, Allis CD (2001). Translating the histone code. Science.

[9] Strahl BD, Allis CD (2000). The language of covalent histone modifications. Nature.

[10] Shi Y, Lan F, Matson C, Mulligan P, Whetstine JR, Cole PA, Casero RA, Shi Y (2004). Histone demethylation mediated by the nuclear amine oxidase homolog LSD1. Cell.

[11] Tsukada Y, Fang J, Erdjument-Bromage H, Warren ME, Borchers CH, Tempst P, Zhang Y (2006). Histone demethylation by a family of JmjC domain-containing proteins. Nature.

[12] Whetstine JR, Nottke A, Lan F, Huarte M, Smolikov S, Chen Z, Spooner E, Li E, Zhang G, Colaiacovo M, Shi Y (2006). Reversal of histone lysine trimethylation by the JMJD2 family of histone demethylases. Cell.

[13] Chen Z, Zang J, Whetstine J, Hong X, Davrazou F, Kutateladze TG, Simpson M, Mao Q, Pan CH, Dai S, Hagman J, Hansen K, Shi Y, Zhang G (2006). Structural insights into histone demethylation by JMJD2 family members. Cell.

[14] Huang RC, Bonner J (1962). Histone, a suppressor of chromosomal RNA synthesis. Proc Natl Acad Sci U S A.

[15] Allfrey VG, Faulkner R, Mirsky AE (1964). Acetylation and methylation of histones and their possible role in the regulation of RNA synthesis. Proc Natl Acad Sci U S A.

[16] Paik WK, Kim S (1967). E-N-dimethyllysine in histones. Biochem Biophys Res Commun.

[17] Hempel K, Lange HW, Birkofer L (1968). [Epsilon-N-trimethyllysine, a new amino acid in histones] [Article in German]. Naturwissenschaften.

[18] Paik WK, Kim S (1968). Protein methylase I. Purification and properties of the enzyme. J Biol Chem.

[19] Baldwin GS, Carnegie PR (1971). Specific enzymic methylation of an arginine in the experimental allergic encephalomyelitis protein from human myelin. Science.

[20] Brostoff S, Eylar EH (1971). Localization of methylated arginine in the A1 protein from myelin. Proc Natl Acad Sci U S A.

[21] Liss M, Edelstein LM (1967). Evidence for the enzymatic methylation of crystalline ovalbumin preparations. Biochem Biophys Res Commun.

[22] Paik WK, Kim S (1970). Solubilization and partial purification of protein methylase 3 from calf thymus nuclei. J Biol Chem.

[23] Paik WK, Kim S (1971). Protein methylation. Science.

[24] McBride AE, Silver PA (2001). State of the arg: protein methylation at arginine comes of age. Cell.

[25] Bedford MT, Richard S (2005). Arginine methylation an emerging regulator of protein function. Mol Cell.

[26] Lee DY, Teyssier C, Strahl BD, Stallcup MR (2005). Role of protein methylation in regulation of transcription. Endocr Rev.

[27] Fackelmayer FO (2005). Protein arginine methyltransferases: guardians of the Arg?. Trends Biochem Sci.

[28] Pahlich S, Zakaryan RP, Gehring H (2006). Protein arginine methylation: cellular functions and methods of analysis. Biochim Biophys Acta.

[29] Sprung R, Chen Y, Zhang K, Cheng D, Zhang T, Peng J, Zhao Y (2008). Identification and validation of eukaryotic aspartate and glutamatemethylation in proteins. J Proteome Res.

[30] Terwilliger TC, Wang JY, Koshland DE (1986). Surface structure recognized for covalent modification of the aspartate receptor in chemotaxis. Proc Natl Acad Sci U S A.

[31] Rice MS, Dahlquist FW (1991). Sites of deamidation and methylation in Tsr, a bacterial chemotaxis sensory transducer. J Biol Chem.

[32] Dunten P, Koshland DE (1991). Tuning the responsiveness of a sensory receptor via covalent modification. J Biol Chem.

[33] Chelsky D, Sobotka C, O’Neill CL (1989). Lamin B methylation and assembly into the nuclear envelope. J Biol Chem.

[34] Sobotka-Briner C, Chelsky D (1992). COOH-terminal methylation of lamin B and inhibition of methylation by farnesylated peptides corresponding to lamin B and other CAAX motif proteins. J Biol Chem.

[35] Huzoor-Akbar Wang W, Kornhauser R, Volker C, Stock JB (1993). Protein prenylcysteine analog inhibits agonist-receptor-mediated signal transduction in human platelets. Proc Natl Acad Sci U S A.

[36] Volker C, Miller RA, McCleary WR, Rao A, Poenie M, Backer JM, Stock JB (1991). Effects of farnesylcysteine analogs on protein carboxyl methylation and signal transduction. J Biol Chem.

[37] Philips MR, Pillinger MH, Staud R, Volker C, Rosenfeld MG, Weissmann G, Stock JB (1993). Carboxyl methylation of Ras-related proteins during signal transduction in neutrophils. Science.

[38] Scheer A, Gierschik P (1993). Farnesylcysteine analogues inhibit chemotactic peptide receptor-mediated G-protein activation in human HL-60 granulocyte membranes. FEBS Lett.

[39] Paik WK, Paik DC, Kim S (2007). Historical review: the field of protein methylation. Trends Biochem Sci.

[40] Solomon LR, Rubenstein PA (1987). Studies on the role of actin’s N tau-methylhistidine using oligodeoxynucleotide-directed site-specific mutagenesis. J Biol Chem.

[41] Swanson RV, Glazer AN (1990). Phycobiliprotein methylation: Effect of the γ-N-methylasparagine residue on energy transfer in phycocyanin and the phycobilisome. J Mol Biol.

[42] Thomas B, Bricker TM, Klotza AV (1993). Post-translational methylation of phycobilisomes and oxygen evolution efficiency in cyanobacteria. Biochim Biophys Acta.

[43] Fisk JC, Read LK (2011). Protein arginine methylation in parasitic protozoa. Eukaryot Cell.

[44] Bachand F (2007). Protein arginine methyltransferases: from unicellular eukaryotes to humans. Eukaryot Cell.

[45] Kouzarides T (2007). Chromatin modifications and their function. Cell.

[46] Bedford MT, Clarke SG (2009). Protein arginine methylation in mammals: who, what, and why. Mol Cell.

[47] Cuthbert GL, Daujat S, Snowden AW, Erdjument-Bromage H, Hagiwara T, Yamada M, Schneider R, Gregory PD, Tempst P, Bannister AJ, Kouzarides T (2004). Histone deimination antagonizes arginine methylation. Cell.

[48] Le Romancer M, Treilleux I, Leconte N, Robin-Lespinasse Y, Sentis S, Bouchekioua-Bouzaghou K, Goddard S, Gobert-Gosse S, Corbo L (2008). Regulation of estrogen rapid signaling through arginine methylation by PRMT1. Mol Cell.

[49] Morales Y, Cáceres T, May K, Hevel JM (2016). Biochemistry and regulation of the protein arginine methyltransferases (PRMTs). Arch Biochem Biophys.

[50] Wang YC, Li C (2012). Evolutionarily conserved protein arginine methyltransferases in non-mammalian animal systems. FEBS J.

[51] Wei H, Mundade R, Lange KC, Lu T (2014). Protein arginine methylation of non-histone proteins and its role in diseases. Cell Cycle.

[52] Di Lorenzo A, Bedford MT (2011). Histone arginine methylation. FEBS Lett.

[53] Kirmizis A, Santos-Rosa H, Penkett CJ, Singer MA, Green RD, Kouzarides T (2009). Distinct transcriptional outputs associated with mono- and dimethylated histone H3 arginine 2. Nat Struct Mol Biol.

[54] Migliori V, Müller J, Phalke S, Low D, Bezzi M, Mok WC, Sahu SK, Gunaratne J, Capasso P, Bassi C, Cecatiello V, De Marco A, Blackstock W (2012). Symmetric dimethylation of H3R2 is a newly identified histone mark that supports euchromatin maintenance. Nat Struct Mol Biol.

[55] Varier RA, Timmers HT (2011). Histone lysine methylation and demethylation pathways in cancer. Biochim Biophys Acta.

[56] Pray-Grant MG, Daniel JA, Schieltz D, Yates JR, Grant PA (2005). Chd1 chromodomain links histone H3 methylation with SAGA- and SLIK-dependent acetylation. Nature.

[57] Huyen Y, Zgheib O, Ditullio RA, Gorgoulis VG, Zacharatos P, Petty TJ, Sheston EA, Mellert HS, Stavridi ES, Halazonetis TD (2004). Methylated lysine 79 of histone H3 targets 53BP1 to DNA double-strand breaks. Nature.

[58] McKenna NJ, Lanz RB, O’Malley BW (1999). Nuclear receptor coregulators: cellular and molecular biology. Endocr Rev.

[59] Rosenfeld MG, Lunyak VV, Glass CK (2006). Sensors and signals: a coactivator/corepressor/epigenetic code for integrating signal-dependent programs of transcriptional response. Genes Dev.

[60] O’Malley BW, Kumar R (2009). Nuclear receptor coregulators in cancer biology. Cancer Res.

[61] Metzger E, Wissmann M, Yin N, Müller JM, Schneider R, Peters AH, Günther T, Buettner R, Schüle R (2005). LSD1 demethylates repressive histone marks to promote androgen-receptor-dependent transcription. Nature.

[62] Wissmann M, Yin N, Müller JM, Greschik H, Fodor BD, Jenuwein T, Vogler C, Schneider R, Günther T, Buettner R, Metzger E, Schüle R (2007). Cooperative demethylation by JMJD2C and LSD1 promotes androgen receptor-dependent gene expression. Nat Cell Biol.

[63] Choi HK, Choi KC, Oh SY, Kang HB, Lee YH, Haam S, Ahn YH, Kim KS, Kim K, Yoon HG (2007). The functional role of the CARM1-SNF5 complex and its associated HMT activity in transcriptional activation by thyroid hormone receptor. Exp Mol Med.

[64] Garcia-Bassets I, Kwon YS, Telese F, Prefontaine GG, Hutt KR, Cheng CS, Ju BG, Ohgi KA, Wang J, Escoubet-Lozach L, Rose DW, Glass CK, Fu XD, Rosenfeld MG (2007). Histone methylation-dependent mechanisms impose ligand dependency for gene activation by nuclear receptors. Cell.

[65] Bhaumik SR, Smith E, Shilatifard A (2007). Covalent modifications of histones during development and disease pathogenesis. Nat Struct Mol Biol.

[66] Anand R, Marmorstein R (2007). Structure and mechanism of lysine-specific demethylase enzymes. J Biol Chem.

[67] Forneris F, Binda C, Battaglioli E, Mattevi A (2008). LSD1: oxidative chemistry for multifaceted functions in chromatin regulation. Trends Biochem Sci.

[68] Karytinos A, Forneris F, Profumo A, Ciossani G, Battaglioli E, Binda C, Mattevi A (2009). A novel mammalian flavin-dependent histone demethylase. J Biol Chem.

[69] Lee MG, Wynder C, Cooch N, Shiekhattar R (2005). An essential role for CoREST in nucleosomal histone 3 lysine 4 demethylation. Nature.

[70] Schulte JH, Lim S, Schramm A, Friedrichs N, Koster J, Versteeg R, Ora I, Pajtler K, Klein-Hitpass L, Kuhfittig-Kulle S, Metzger E, Schüle R, Eggert A (2009). Lysine-specific demethylase 1 is strongly expressed in poorly differentiated neuroblastoma: implications for therapy. Cancer Res.

[71] Hu X, Li X, Valverde K, Fu X, Noguchi C, Qiu Y, Huang S (2009). LSD1-mediated epigenetic modification is required for TAL1 function and hematopoiesis. Proc Natl Acad Sci U S A.

[72] Gu H, Roizman B (2009). Engagement of the lysine-specific demethylase/HDAC1/CoREST/REST complex by herpes simplex virus 1. J Virol.

[73] Ciccone DN, Su H, Hevi S, Gay F, Lei H, Bajko J, Xu G, Li E, Chen T (2009). KDM1B is a histone H3K4 demethylase required to establish maternal genomic imprints. Nature.

[74] Yang Z, Jiang J, Stewart MD, Qi S, Yamane K, Li J, Zhang Y, Wong J (2010). AOF1 is a histone H3K4 demethylase possessing demethylase activity-independent repression function. Cell Res.

[75] Edmondson DE, Binda C, Wang J, Upadhyay AK, Mattevi A (2009). Molecular and mechanistic properties of the membrane-bound mitochondrial monoamine oxidases. Biochemistry.

[76] Shi YJ, Matson C, Lan F, Iwase S, Baba T, Shi Y (2005). Regulation of LSD1 histone demethylase activity by its associated factors. Mol Cell.

[77] Fang R, Barbera AJ, Xu Y, Rutenberg M, Leonor T, Bi Q, Lan F, Mei P, Yuan GC, Lian C, Peng J, Cheng D, Sui G (2010). Human LSD2/KDM1b/AOF1 regulates gene transcription by modulating intragenic H3K4me2 methylation. Mol Cell.

[78] van Essen D, Zhu Y, Saccani S (2010). A feed-forward circuit controlling inducible NF-κB target gene activation by promoter histone demethylation. Mol Cell.

[79] Core LJ, Lis JT (2008). Transcription regulation through promoter-proximal pausing of RNA polymerase II. Science.

[80] Yang M, Gocke CB, Luo X, Borek D, Tomchick DR, Machius M, Otwinowski Z, Yu H (2006). Structural basis for CoREST-dependent demethylation of nucleosomes by the human LSD1 histone demethylase. Mol Cell.

[81] Yang M, Culhane JC, Szewczuk LM, Gocke CB, Brautigam CA, Tomchick DR, Machius M, Cole PA, Yu H (2007). Structural basis of histone demethylation by LSD1 revealed by suicide inactivation. Nat Struct Mol Biol.

[82] He F, Umehara T, Saito K, Harada T, Watanabe S, Yabuki T, Kigawa T, Takahashi M, Kuwasako K, Tsuda K, Matsuda T, Aoki M, Seki E (2010). Structural insight into the zinc finger CW domain as a histone modification reader. Structure.

[83] Hoppmann V, Thorstensen T, Kristiansen PE, Veiseth SV, Rahman MA, Finne K, Aalen RB, Aasland R (2011). The CW domain, a new histone recognition module in chromatin proteins. EMBO J.

[84] Ballas N, Battaglioli E, Atouf F, Andres ME, Chenoweth J, Anderson ME, Burger C, Moniwa M, Davie JR, Bowers WJ, Federoff HJ, Rose DW, Rosenfeld MG (2001). Regulation of neuronal traits by a novel transcriptional complex. Neuron.

[85] Hakimi MA, Bochar DA, Chenoweth J, Lane WS, Mandel G, Shiekhattar R (2002). A core-BRAF35 complex containing histone deacetylase mediates repression of neuronal-specific genes. Proc Natl Acad Sci U S A.

[86] Hakimi MA, Dong Y, Lane WS, Speicher DW, Shiekhattar R (2003). A candidate X-linked mental retardation gene is a component of a new family of histone deacetylase-containing complexes. J Biol Chem.

[87] Humphrey GW, Wang Y, Russanova VR, Hirai T, Qin J, Nakatani Y, Howard BH (2001). Stable histone deacetylase complexes distinguished by the presence of SANT domain proteins CoREST/kiaa0071 and Mta-L1. J Biol Chem.

[88] Shi Y, Sawada J, Sui G, Affar B, Whetstine JR, Lan F, Ogawa H, Luke MP, Nakatani Y, Shi Y (2003). Coordinated histone modifications mediated by a CtBP co-repressor complex. Nature.

[89] You A, Tong JK, Grozinger CM, Schreiber SL (2001). CoREST is an integral component of the CoREST- human histone deacetylase complex. Proc Natl Acad Sci U S A.

[90] Holbert MA, Marmorstein R (2005). Structure and activity of enzymes that remove histone modifications. Curr Opin Struct Biol.

[91] Fraaije MW, Van Berkel WJ, Benen JA, Visser J, Mattevi A (1998). A novel oxidoreductase family sharing a conserved FAD-binding domain. Trends Biochem Sci.

[92] Fraaije MW, Mattevi A (2000). Flavoenzymes: diverse catalysts with recurrent features. Trends Biochem Sci.

[93] Aravind L, Iyer LM (2002). The SWIRM domain: a conserved module found in chromosomal proteins points to novel chromatin-modifying activities. Genome Biol.

[94] Chen Y, Yang Y, Wang F, Wan K, Yamane K, Zhang Y, Lei M (2006). Crystal structure of human histone lysine-specific demethylase 1 (LSD1). Proc Natl Acad Sci U S A.

[95] Forneris F, Binda C, Adamo A, Battaglioli E, Mattevi A (2007). Structural basis of LSD1-CoREST selectivity in histone H3 recognition. J Biol Chem.

[96] Kahl P, Gullotti L, Heukamp LC, Wolf S, Friedrichs N, Vorreuther R, Solleder G, Bastian PJ, Ellinger J, Metzger E, Schüle R, Buettner R (2006). Androgen receptor coactivators lysine-specific histone demethylase 1 and four and a half LIM domain protein 2 predict risk of prostate cancer recurrence. Cancer Res.

[97] Wang J, Scully K, Zhu X, Cai L, Zhang J, Prefontaine GG, Krones A, Ohgi KA, Zhu P, Garcia-Bassets I, Liu F, Taylor H, Lozach J (2007). Opposing LSD1 complexes function in developmental gene activation and repression programmes. Nature.

[98] Zhu Q, Liu C, Ge Z, Fang X, Zhang X, Strååt K, Björkholm M, Xu D (2008). Lysine-specific demethylase 1 (LSD1) Is required for the transcriptionalrepression of the telomerase reverse transcriptase (hTERT) gene. PLoS One.

[99] Huang J, Sengupta R, Espejo AB, Lee MG, Dorsey JA, Richter M, Opravil S, Shiekhattar R, Bedford MT, Jenuwein T, Berger SL (2007). p53 is regulated by the lysine demethylase LSD1. Nature.

[100] Lin Y, Wu Y, Li J, Dong C, Ye X, Chi YI, Evers BM, Zhou BP (2010). The SNAG domain of Snail1 functions as a molecular hook for recruiting lysine-specific demethylase 1. EMBO J.

[101] Hu Q, Kwon YS, Nunez E, Cardamone MD, Hutt KR, Ohgi KA, Garcia-Bassets I, Rose DW, Glass CK, Rosenfeld MG, Fu XD (2008). Enhancing nuclear receptor-induced transcription requires nuclear motor and LSD1-dependent gene networking in interchromatin granules. Proc Natl Acad Sci U S A.

[102] Lim S, Janzer A, Becker A, Zimmer A, Schüle R, Buettner R, Kirfel J (2010). Lysine-specific demethylase 1 (LSD1) is highly expressed in ER-negative breast cancers and a biomarker predicting aggressive biology. Carcinogenesis.

[103] Chen J, Ding J, Wang Z, Zhu J, Wang X, Du J (2017). Identification of downstream metastasis-associated target genes regulated by LSD1 in colon cancer cells. Oncotarget.

[104] Takawa M, Masuda K, Kunizaki M, Daigo Y, Takagi K, Iwai Y, Cho HS, Toyokawa G, Yamane Y, Maejima K, Field HI, Kobayashi T, Akasu T (2011). Validation of the histone methyltransferase EZH2 as a therapeutic target for various types of human cancer and as a prognostic marker. Cancer Sci.

[105] Sasaki M, Yamaguchi J, Itatsu K, Ikeda H, Nakanuma Y (2008). Over-expression of polycomb group protein EZH2 relates to decreased expression of p16 INK4a in cholangiocarcinogenesis in hepatolithiasis. J Pathol.

[106] Hamamoto R, Furukawa Y, Morita M, Iimura Y, Silva FP, Li M, Yagyu R, Nakamura Y (2004). SMYD3 encodes a histone methyltransferase involved in the proliferation of cancer cells. Nat Cell Biol.

[107] Hamamoto R, Silva FP, Tsuge M, Nishidate T, Katagiri T, Nakamura Y, Furukawa Y (2006). Enhanced SMYD3 expression is essential for the growth of breast cancer cells. Cancer Sci.

[108] Cho HS, Kelly JD, Hayami S, Toyokawa G, Takawa M, Yoshimatsu M, Tsunoda T, Field HI, Neal DE, Ponder BA, Nakamura Y, Hamamoto R (2011). Enhanced expression of EHMT2 is involved in the proliferation of cancer cells through negative regulation of SIAH1. Neoplasia.

[109] Cho HS, Toyokawa G, Daigo Y, Hayami S, Masuda K, Ikawa N, Yamane Y, Maejima K, Tsunoda T, Field HI, Kelly JD, Neal DE, Ponder BA (2012). The JmjC domain-containing histone demethylase KDM3A is a positive regulator of the G1/S transition in cancer cells via transcriptional regulation of the HOXA1 gene. Int J Cancer.

[110] Toyokawa G, Cho HS, Iwai Y, Yoshimatsu M, Takawa M, Hayami S, Maejima K, Shimizu N, Tanaka H, Tsunoda T, Field HI, Kelly JD, Neal DE (2011). The histone demethylase JMJD2B plays an essential role in human carcinogenesis through positive regulation of cyclin-dependent kinase 6. Cancer Prev Res (Phila).

[111] Smith BC, Denu JM (2009). Chemical mechanisms of histone lysine and arginine modifications. Biochim Biophys Acta.

[112] Feng Q, Wang H, Ng HH, Erdjument-Bromage H, Tempst P, Struhl K, Zhang Y (2002). Methylation of H3-lysine 79 is mediated by a new family of HMTases without a SET domain. Curr Biol.

[113] Shimazu T, Barjau J, Sohtome Y, Sodeoka M, Shinkai Y (2014). Selenium-based S-adenosylmethionine analog reveals the mammalian seven-beta-strand methyltransferase METTL10 to be an EF1A1 lysinemethyltransferase. PLoS One.

[114] Hayami S, Kelly JD, Cho HS, Yoshimatsu M, Unoki M, Tsunoda T, Field HI, Neal DE, Yamaue H, Ponder BA, Nakamura Y, Hamamoto R (2011). Overexpression of LSD1 contributes to human carcinogenesis through chromatin regulation in various cancers. Int J Cancer.

[115] Kauffman EC, Robinson BD, Downes MJ, Powell LG, Lee MM, Scherr DS, Gudas LJ, Mongan NP (2011). Role of androgen receptor and associated lysine-demethylase coregulators, LSD1 and JMJD2A, in localized and advanced human bladder cancer. Mol Carcinog.

[116] van Haaften G, Dalgliesh GL, Davies H, Chen L, Bignell G, Greenman C, Edkins S, Hardy C, O’Meara S, Teague J, Butler A, Hinton J, Latimer C (2009). Somatic mutations of the histone H3K27 demethylase gene UTX in human cancer. Nat Genet.

[117] Dalgliesh GL, Furge K, Greenman C, Chen L, Bignell G, Butler A, Davies H, Edkins S, Hardy C, Latimer C, Teague J, Andrews J, Barthorpe S (2010). Systematic sequencing of renal carcinoma reveals inactivation of histone modifying genes. Nature.

[118] Varambally S, Dhanasekaran SM, Zhou M, Barrette TR, Kumar-Sinha C, Sanda MG, Ghosh D, Pienta KJ, Sewalt RG, Otte AP, Rubin MA, Chinnaiyan AM (2002). The polycomb group protein EZH2 is involved in progression of prostate cancer. Nature.

[119] Morin RD, Johnson NA, Severson TM, Mungall AJ, An J, Goya R, Paul JE, Boyle M, Woolcock BW, Kuchenbauer F, Yap D, Humphries RK, Griffith OL (2010). Somatic mutations altering EZH2 (Tyr641) in follicular and diffuse large B-cell lymphomas of germinal-center origin. Nat Genet.

[120] Harris WJ, Huang X, Lynch JT, Spencer GJ, Hitchin JR, Li Y, Ciceri F, Blaser JG, Greystoke BF, Jordan AM, Miller CJ, Ogilvie DJ, Somervaille TC (2012). The histone demethylase KDM1A sustains the oncogenic potential of MLL-AF9 leukemia stem cells. Cancer Cell.

[121] Schenk T, Chen WC, Göllner S, Howell L, Jin L, Hebestreit K, Klein HU, Popescu AC, Burnett A, Mills K, Casero RA, Marton L, Woster P (2012). Inhibition of the LSD1 (KDM1A) demethylase reactivates the all-trans-retinoic acid differentiation pathway in acute myeloid leukemia. Nat Med.

[122] Liu G, Bollig-Fischer A, Kreike B, van de Vijver MJ, Abrams J, Ethier SP, Yang ZQ (2009). Genomic amplification and oncogenic properties of the GASC1 histone demethylase gene in breast cancer. Oncogene.

[123] Ehrbrecht A, Müller U, Wolter M, Hoischen A, Koch A, Radlwimmer B, Actor B, Mincheva A, Pietsch T, Lichter P, Reifenberger G, Weber RG (2006). Comprehensive genomic analysis of desmoplastic medulloblastomas: identification of novel amplified genes and separate evaluation of the different histological components. J Pathol.

[124] Shi L, Sun L, Li Q, Liang J, Yu W, Yi X, Yang X, Li Y, Han X, Zhang Y, Xuan C, Yao Z, Shang Y (2011). Histone demethylase JMJD2B coordinates H3K4/H3K9 methylation and promotes hormonally responsive breast carcinogenesis. Proc Natl Acad Sci U S A.

[125] Kawazu M, Saso K, Tong KI, McQuire T, Goto K, Son DO, Wakeham A, Miyagishi M, Mak TW, Okada H (2011). Histone demethylase JMJD2B functions as a co-factor of estrogen receptor in breast cancer proliferation and mammary gland development. PLoS One.

[126] Yang J, Jubb AM, Pike L, Buffa FM, Turley H, Baban D, Leek R, Gatter KC, Ragoussis J, Harris AL (2010). The histone demethylase JMJD2B is regulated by estrogen receptor alpha and hypoxia, and is a key mediator of estrogen induced growth. Cancer Res.

[127] Fu L, Chen L, Yang J, Ye T, Chen Y, Fang J (2012). HIF-1α-induced histone demethylase JMJD2B contributes to the malignant phenotype of colorectal cancer cells via an epigenetic mechanism. Carcinogenesis.

[128] Peters AH, O’Carroll D, Scherthan H, Mechtler K, Sauer S, Schöfer C, Weipoltshammer K, Pagani M, Lachner M, Kohlmaier A, Opravil S, Doyle M, Sibilia M, Jenuwein T (2001). Loss of the Suv39h histone methyltransferases impairs mammalian heterochromatin and genome stability. Cell.

[129] Braig M, Lee S, Loddenkemper C, Rudolph C, Peters AH, Schlegelberger B, Stein H, Dörken B, Jenuwein T, Schmitt CA (2005). Oncogene-induced senescence as an initial barrier in lymphoma development. Nature.

[130] Cloos PA, Christensen J, Agger K, Maiolica A, Rappsilber J, Antal T, Hansen KH, Helin K (2006). The putative oncogene GASC1 demethylates tri- and dimethylated lysine 9 on histone H3. Nature.

[131] Rui L, Emre NC, Kruhlak MJ, Chung HJ, Steidl C, Slack G, Wright GW, Lenz G, Ngo VN, Shaffer AL, Xu W, Zhao H, Yang Y (2010). Cooperative epigenetic modulation by cancer amplicon genes. Cancer Cell.

[132] Albert M, Schmitz SU, Kooistra SM, Malatesta M, Morales Torres C, Rekling JC, Johansen JV, Abarrategui I, Helin K (2013). The histone demethylase Jarid1b ensures faithful mouse development by protecting developmental genes from aberrant H3K4me3. PLoS Genet.

[133] Schmitz SU, Albert M, Malatesta M, Morey L, Johansen JV, Bak M, Tommerup N, Abarrategui I, Helin K (2011). Jarid1b targets genes regulating development and is involved in neural differentiation. EMBO J.

[134] Hayami S, Yoshimatsu M, Veerakumarasivam A, Unoki M, Iwai Y, Tsunoda T, Field HI, Kelly JD, Neal DE, Yamaue H, Ponder BA, Nakamura Y, Hamamoto R (2010). Overexpression of the JmjC histone demethylase KDM5B in human carcinogenesis: involvement in the proliferation of cancer cells through the E2F/RB pathway. Mol Cancer.

[135] Xiang Y, Zhu Z, Han G, Ye X, Xu B, Peng Z, Ma Y, Yu Y, Lin H, Chen AP, Chen CD (2007). JARID1B is a histone H3 lysine 4 demethylase up-regulated in prostate cancer. Proc Natl Acad Sci U S A.

[136] Yamane K, Tateishi K, Klose RJ, Fang J, Fabrizio LA, Erdjument-Bromage H, Taylor-Papadimitriou J, Tempst P, Zhang Y (2007). PLU-1 is an H3K4 demethylase involved in transcriptional repression and breast cancer cell proliferation. Mol Cell.

[137] Roesch A, Fukunaga-Kalabis M, Schmidt EC, Zabierowski SE, Brafford PA, Vultur A, Basu D, Gimotty P, Vogt T, Herlyn M (2010). A temporarily distinct subpopulation of slow-cycling melanoma cells is required for continuous tumor growth. Cell.

[138] Wu X, Johansen JV, Helin K (2013). Fbxl10/Kdm2b recruits polycomb repressive complex 1 to CpG islands and regulates H2A ubiquitylation. Mol Cell.

[139] He J, Nguyen AT, Zhang Y (2011). KDM2b/JHDM1b, an H3K36me2-specific demethylase, is required for initiation and maintenance of acute myeloid leukemia. Blood.

[140] Tzatsos A, Paskaleva P, Ferrari F, Deshpande V, Stoykova S, Contino G, Wong KK, Lan F, Trojer P, Park PJ, Bardeesy N (2013). KDM2B promotes pancreatic cancer via Polycomb-dependent and -independent transcriptional programs. J Clin Invest.

[141] Farcas AM, Blackledge NP, Sudbery I, Long HK, McGouran JF, Rose NR, Lee S, Sims D, Cerase A, Sheahan TW, Koseki H, Brockdorff N, Ponting CP (2012). KDM2B links the Polycomb Repressive Complex 1 (PRC1) to recognition of CpG islands. eLife.

[142] Su ST, Ying HY, Chiu YK, Lin FR, Chen MY, Lin KI (2009). Involvement of histone demethylase LSD1 in Blimp-1-mediated gene repression during plasma cell differentiation. Mol Cell Biol.

[143] Yokoyama A, Takezawa S, Schüle R, Kitagawa H, Kato S (2008). Transrepressive function of TLX requires the histone demethylase LSD1. Mol Cell Biol.

[144] Wong CM, Ng YL, Lee JM, Wong CC, Cheung OF, Chan CY, Tung EK, Ching YP, Ng IO (2007). Tissue factor pathway inhibitor-2 as a frequently silenced tumor suppressor gene in hepatocellular carcinoma. Hepatology.

[145] Wang J, Hevi S, Kurash JK, Lei H, Gay F, Bajko J, Su H, Sun W, Chang H, Xu G, Gaudet F, Li E, Chen T (2009). The lysine demethylase LSD1 (KDM1) is required for maintenance of global DNA methylation. Nat Genet.

[146] Saleque S, Kim J, Rooke HM, Orkin SH (2007). Epigenetic regulation of hematopoietic differentiation by Gfi-1 and Gfi-1b is mediated by the cofactors CoREST and LSD1. Mol Cell.

[147] Kogure M, Takawa M, Saloura V, Sone K, Piao L, Ueda K, Ibrahim R, Tsunoda T, Sugiyama M, Atomi Y, Nakamura Y, Hamamoto R (2013). The oncogenic polycomb histone methyltransferase EZH2 methylates lysine 120 on histone H2B and competes ubiquitination. Neoplasia.

[148] Sone K, Piao L, Nakakido M, Ueda K, Jenuwein T, Nakamura Y, Hamamoto R (2014). Critical role of lysine 134 methylation on histone H2AX for γ-H2AX production and DNA repair. Nat Commun.

[149] Mazur PK, Reynoird N, Khatri P, Jansen PW, Wilkinson AW, Liu S, Barbash O, Van Aller GS, Huddleston M, Dhanak D, Tummino PJ, Kruger RG, Garcia BA (2014). SMYD3 links lysine methylation of MAP3K2 to Ras-driven cancer. Nature.

[150] Khorasanizadeh S (2011). Recognition of methylated histones: new twists and variations. Curr Opin Struct Biol.

[151] Daze KD, Hof F (2013). The cation-π interaction at protein-protein interaction interfaces: developing and learning from synthetic mimics of proteins that bind methylated lysines. Acc Chem Res.

[152] Pang CN, Gasteiger E, Wilkins MR (2010). Identification of arginine- and lysine-methylation in the proteome of Saccharomyces cerevisiae and its functional implications. BMC Genomics.

[153] Lee JM, Lee JS, Kim H, Kim K, Park H, Kim JY, Lee SH, Kim IS, Kim J, Lee M, Chung CH, Seo SB, Yoon JB (2012). EZH2 generates a methyl degron that is recognized by the DCAF1/DDB1/CUL4 E3 ubiquitin ligase complex. Mol Cell.

[154] Hodel MR, Corbett AH, Hodel AE (2001). Dissection of a nuclear localization signal. J Biol Chem.

[155] Cho HS, Shimazu T, Toyokawa G, Daigo Y, Maehara Y, Hayami S, Ito A, Masuda K, Ikawa N, Field HI, Tsuchiya E, Ohnuma S, Ponder BA (2012). Enhanced HSP70 lysine methylation promotes proliferation of cancer cells through activation of Aurora kinase. B. Nat Commun.

[156] Chuikov S, Kurash JK, Wilson JR, Xiao B, Justin N, Ivanov GS, McKinney K, Tempst P, Prives C, Gamblin SJ, Barlev NA, Reinberg D (2004). Regulation of p53 activity through lysine methylation. Nature.

[157] Stark GR, Wang Y, Lu T (2011). Lysine methylation of promoter-bound transcription factors and relevance to cancer. Cell Res.

[158] Lu T, Jackson MW, Wang B, Yang M, Chance MR, Miyagi M, Gudkov AV, Stark GR (2010). Regulation of NF-kappaB by NSD1/FBXL11-dependent reversible lysine methylation of p65. Proc Natl Acad Sci U S A.

[159] Lu T, Yang M, Huang DB, Wei H, Ozer GH, Ghosh G, Stark GR (2013). Role of lysine methylation of NF-κB in differential gene regulation. Proc Natl Acad Sci U S A.

[160] Greiner D, Bonaldi T, Eskeland R, Roemer E, Imhof A (2005). Identification of a specific inhibitor of the histone methyltransferase SU(VAR)3-9. Nat Chem Biol.

[161] Daigle SR, Olhava EJ, Therkelsen CA, Majer CR, Sneeringer CJ, Song J, Johnston LD, Scott MP, Smith JJ, Xiao Y, Jin L, Kuntz KW, Chesworth R (2011). Selective killing of mixed lineage leukemia cells by a potent small-molecule DOT1L inhibitor. Cancer Cell.

[162] Jo SY, Granowicz EM, Maillard I, Thomas D, Hess JL (2011). Requirement for Dot1l in murine postnatal hematopoiesis and leukemogenesis by MLL translocation. Blood.

[163] Kubicek S, O’Sullivan RJ, August EM, Hickey ER, Zhang Q, Teodoro ML, Rea S, Mechtler K, Kowalski JA, Homon CA, Kelly TA, Jenuwein T (2007). Reversal of H3K9me2 by a small-molecule inhibitor for the G9a histone methyltransferase. Mol Cell.

[164] McCabe MT, Ott HM, Ganji G, Korenchuk S, Thompson C, Van Aller GS, Liu Y, Graves AP, Della Pietra A, Diaz E, LaFrance LV, Mellinger M, Duquenne C (2012). EZH2 inhibition as a therapeutic strategy for lymphoma with EZH2-activating mutations. Nature.

[165] Knutson SK, Wigle TJ, Warholic NM, Sneeringer CJ, Allain CJ, Klaus CR, Sacks JD, Raimondi A, Majer CR, Song J, Scott MP, Jin L, Smith JJ (2012). A selective inhibitor of EZH2 blocks H3K27 methylation and kills mutant lymphoma cells. Nat Chem Biol.

[166] Knutson SK, Kawano S, Minoshima Y, Warholic NM, Huang KC, Xiao Y, Kadowaki T, Uesugi M, Kuznetsov G, Kumar N, Wigle TJ, Klaus CR, Allain CJ (2014). Selective inhibition of EZH2 by EPZ-6438 leads to potent antitumor activity in EZH2-mutant non-Hodgkin lymphoma. Mol Cancer Ther.

[167] Basavapathruni A, Olhava EJ, Daigle SR, Therkelsen CA, Jin L, Boriack-Sjodin PA, Allain CJ, Klaus CR, Raimondi A, Scott MP, Dovletoglou A, Richon VM, Pollock RM (2014). Nonclinical pharmacokinetics and metabolism of EPZ-5676, a novel DOT1L histone methyltransferase inhibitor. Biopharm Drug Dispos.

[168] Klaus CR, Iwanowicz D, Johnston D, Campbell CA, Smith JJ, Moyer MP, Copeland RA, Olhava EJ, Scott MP, Pollock RM, Daigle SR, Raimondi A (2014). DOT1L inhibitor EPZ-5676 displays synergistic antiproliferative activity in combination with standard of care drugs and hypomethylating agents in MLL-rearranged leukemia cells. J Pharmacol Exp Ther.

[169] Lee MG, Wynder C, Schmidt DM, McCafferty DG, Shiekhattar R (2006). Histone H3 lysine 4 demethylation is a target of nonselective antidepressive medications. Chem Biol.

[170] Culhane JC, Wang D, Yen PM, Cole PA (2010). Comparative analysis of small molecules and histone substrate analogues as LSD1 lysine demethylase inhibitors. J Am Chem Soc.

[171] Schmidt DM, McCafferty DG (2007). trans-2-Phenylcyclopropylamine is a mechanism-based inactivator of the histone demethylase LSD1. Biochemistry.

[172] Benelkebir H, Hodgkinson C, Duriez PJ, Hayden AL, Bulleid RA, Crabb SJ, Packham G, Ganesan A (2011). Enantioselective synthesis of tranylcypromine analogues as lysine demethylase (LSD1) inhibitors. Bioorg Med Chem.

[173] Culhane JC, Szewczuk LM, Liu X, Da G, Marmorstein R, Cole PA (2006). A mechanism-based inactivator for histone demethylase LSD1. J Am Chem Soc.

[174] Huang Y, Greene E, Murray Stewart T, Goodwin AC, Baylin SB, Woster PM, Casero RA (2007). Inhibition of lysine-specific demethylase 1 by polyamine analogues results in reexpression of aberrantly silenced genes. Proc Natl Acad Sci U S A.

[175] Huang Y, Stewart TM, Wu Y, Baylin SB, Marton LJ, Perkins B, Jones RJ, Woster PM, Casero RA (2009). Novel oligoamine analogues inhibit lysine-specific demethylase 1 and induce reexpression of epigenetically silenced genes. Clin Cancer Res.

[176] Zhu Q, Huang Y, Marton LJ, Woster PM, Davidson NE, Casero RA (2012). Polyamine analogs modulate gene expression by inhibiting lysine-specific demethylase 1 (LSD1) and altering chromatin structure in human breast cancer cells. Amino Acids.

[177] Vannini A, Volpari C, Filocamo G, Casavola EC, Brunetti M, Renzoni D, Chakravarty P, Paolini C, De Francesco R, Gallinari P, Steinkühler C, Di Marco S (2004). Crystal structure of a eukaryotic zinc-dependent histone deacetylase, human HDAC8, complexed with a hydroxamic acid inhibitor. Proc Natl Acad Sci U S A.

[178] Rose NR, Ng SS, Mecinović J, Liénard BM, Bello SH, Sun Z, McDonough MA, Oppermann U, Schofield CJ (2008). Inhibitor scaffolds for 2-oxoglutarate-dependent histone lysine demethylases. J Med Chem.

[179] Kruidenier L, Chung CW, Cheng Z, Liddle J, Che K, Joberty G, Bantscheff M, Bountra C, Bridges A, Diallo H, Eberhard D, Hutchinson S, Jones E (2012). A selective jumonji H3K27 demethylase inhibitor modulates the proinflammatory macrophage response. Nature.

[180] Wang L, Chang J, Varghese D, Dellinger M, Kumar S, Best AM, Ruiz J, Bruick R, Peña-Llopis S, Xu J, Babinski DJ, Frantz DE, Brekken RA (2013). A small molecule modulates Jumonji histone demethylase activity and selectively inhibits cancer growth. Nat Commun.

